# Optimization of ex vivo expansion of HER2 specific polyfunctional Th1/Th17 cells from HER2 vaccine primed PBMC

**DOI:** 10.1186/2051-1426-1-S1-P5

**Published:** 2013-11-07

**Authors:** Yushe Dang, Lupe Salazar, Jennifer Childs, Doreen Higgins, Mary L Disis

**Affiliations:** 1 Tumor Vaccine Group, Center for Translational Medicine in Women’s Health, University of Washington, Seattle, WA, USA

## 

Adoptive transfer of ex vivo expanded neu specific polyfunctional T-cells secreting TNF-alpha (α), IFN-gamma (γ), and IL-17 (Th1/Th17) cells into tumor bearing mice can result in complete resolution of disease as compared to the use of neu specific Th1 (Lai et al 2009). Murine antigen specific Th1/Th17 cells could be readily expanded with IL-2 and IL-21 in culture, however, the use of these cytokines resulted in successful expansion of human tumor antigen specific T-cells in only a minority of patients. We sought to identify ex vivo culture conditions that would be suitable for the clinical expansion of polyfunctional HER2 specific Th1/Th17 for therapeutic infusion. PBMC, derived from the aphaeresis of patients previously immunized with a HER2 vaccine, were stimulated with HER2 peptides in the presence of different cytokines to polarize Th17 cells, and then cultured with different T-cell growth factors on Day4/8, and subsequently expanded with CD3/CD28 beads on Day 12 and IL-2 for 12 days. We found that IL-1beta (β)/IL-6 generated higher number of IL-17 secreting CD4 cells before CD3/CD28 activation. Other cytokine combinations, including IL-1β/IL-6/IL-21, IL-1β/IL-6/anti-TGFβ antibody, and IL-21 alone, failed to further increase IL-17 cells. A low dose of IL-2 alone added in the culture on Day 4/8, following HER2 peptide and IL-1β/IL-6, generated a higher number of antigen specific IL-17 secreting cells than the combinations of IL-2/IL-7 and IL-2/IL-7/IL-15. In addition, exposure to IL1-β/IL-6 at the time of antigen stimulation was superior to the cytokines added on Day 4/8. Flow cytometric studies of the T-cells generated showed the generation of a Th1/Th17 phenotype, including dual secreting IL-17 and TNF-α, IL-17 and IFN-γ, and triple secreting IL-17, IFN-γ and TNF-α. These data demonstrate a streamlined methodology, easily adaptable to the clinic, for the generation of tumor specific polyfunctional T-cells for therapeutic infusion.

